# Lipid remodeling under acidic conditions and its interplay with low Pi stress in Arabidopsis

**DOI:** 10.1007/s11103-019-00891-1

**Published:** 2019-06-14

**Authors:** Masato Murakawa, Hiroyuki Ohta, Mie Shimojima

**Affiliations:** School of Life Science and Technology, Tokyo Institute of Technology, 4259-B-65 Nagatsuta-cho, Midori-ku, Yokohama, Kanagawa 226-8501 Japan

**Keywords:** Galactolipid, Digalactosyldiacylglycerol (DGDG), MGDG synthase (MGD), Lipid remodeling, Phosphate starvation, Acidic stress

## Abstract

**Key message:**

Here we show that accumulation of galactose-containing lipids in plastid membranes in shoots and the other membranes in roots maintains *Arabidopsis* growth under acidic stress and acidic phosphate deficiency.

**Abstract:**

Soil acidification and phosphate deficiency are closely related to each other in natural environments. In addition to the toxicity of high proton concentrations, acid soil can lead to imbalances of ion availability and nutritional deficiencies, including inorganic phosphate (Pi). Among plants, activation of non-phosphorus-containing galactolipid, digalactosyldiacylglycerol (DGDG), synthesis concomitant with phospholipid degradation, namely membrane lipid remodeling, is crucial for coping with Pi starvation. However, regulation mechanisms of membrane lipid composition during acidic stress have not been clarified. Here, we investigated lipid metabolism in *Arabidopsis thaliana* grown under acidic stress with or without Pi. Under Pi-sufficient acidic conditions, DGDG was increased in shoot membranes, and some Pi starvation–responsive genes that are involved in lipid remodeling were upregulated without reducing Pi content in leaves. In contrast, under acidic Pi deficiency, membrane lipid remodeling in roots was partially repressed at a lower external pH. Nevertheless, phenotypic comparison between wild type and the double mutant of MGD2/3, which are responsible for DGDG accumulation during Pi starvation, indicated that the complete absence of lipid remodeling in roots resulted in a loss of tolerance to Pi deficiency rather specifically under acidic conditions. This result suggested important physiological roles of galactolipid-enriched membranes under acidic Pi deficiency.

**Electronic supplementary material:**

The online version of this article (10.1007/s11103-019-00891-1) contains supplementary material, which is available to authorized users.

## Introduction

Soil acidification is a major agricultural problem that affects plant growth on a worldwide scale (von Uexküll and Mutert 1995; Shavrukov and Hirai 2016). In acid soils, which are defined as having a pH below 5.0–5.5, an increase in the concentration of protons can limit the growth of plants and also causes other associated stresses; an elution of toxic ions such as aluminum (Al) and manganese from the soil, a decrease in available inorganic phosphate (Pi), molybdenum, calcium, potassium, and magnesium. Pi bioavailability in the soil is often limited by forming insoluble salts with iron or Al, especially in acid soils. Inversely, rhizosphere acidification caused by plants has been reported to occur under Pi deficiency, most likely because of exudation of protons and organic acids and/or imbalances in the uptake of ions (Hinsinger et al. 2003). Thus, for plants in nature, acidic environment is highly associated with Pi deficiency.

Low-pH conditions can irreversibly inhibit the root growth of plants both quickly and severely because of the toxicity of the protons themselves (Koyama et al. 2001). As regards the acidic stress tolerance of plants, during the early phase of exposure to low-pH environments, membrane-localized proton transporters (H^+^-ATPases) are activated and pump out excess protons from the cytoplasm to the extracellular space or sequester them in vacuoles (Yan et al. 1998). With longer-term exposure, detoxification systems for reactive oxygen species and signaling in response to biotic and abiotic stresses, including defense-associated hormone signaling, are induced (Lager et al. 2010; Hachiya et al. 2014). In addition, auxin signaling also contributes to the adaptation to acidic stress through activating a H^+^-ATPase localized in the plasma membrane (Inoue et al. 2016). Although membranes are thought to be impaired by the lipid peroxidation during acidic stress (Zhang et al. 2015), the membrane lipid composition of plants grown under acidic conditions has not yet been analyzed in detail.

Upon Pi starvation, plants alter the membrane lipid composition, called membrane lipid remodeling. To provide Pi for essential biological processes, a substantial portion of phospholipids is degraded and replaced by non-phosphorus lipids (Härtel et al. 2000; Nakamura 2013). In seed plants, Pi starvation enhances accumulation of digalactosyldiacylglycerol (DGDG), which is synthesized on the plastidial envelope membranes and is transported to the extraplastidial membranes: the plasma membrane, mitochondrial membrane, and tonoplast membranes (Andersson et al. 2003, 2005; Jouhet et al. 2004). Under Pi-sufficient conditions, however, galactolipids mainly exist in the plastids. Two kinds of galactolipids, DGDG and monogalactosyldiacylglycerol (MGDG), constitute 26 and 52% of thylakoid membrane from spinach chloroplasts, respectively (Block et al. 1983). These galactolipids are synthesized by MGDG synthases (MGDs) and DGDG synthases (DGDs). MGD transfers a galactosyl moiety from UDP-galactose to diacylglycerol to form MGDG. Subsequently, DGD synthesizes DGDG by galactosylation of MGDG using UDP-galactose as the substrate. *Arabidopsis* has three MGDs and two DGDs, MGD1/2/3 and DGD1/2, respectively (Benning and Ohta 2005). The three MGDs are generally classified as type-A (MGD1) and type-B (MGD2/3) (Miège et al. 1999; Awai et al. 2001). The type-A MGD is localized on the inner envelope membrane of plastids and functions together with DGD1; the MGD1-DGD1 pathway is carried out mainly in green tissues and is responsible for the accumulation of the bulk of galactolipids in chloroplast membranes. In the shoots of *Arabidopsis* MGD1 knock-out mutant, the amount of MGDG was reduced by ~ 98% compared with the wild-type (WT), and DGDG was also scarcely detected (Kobayashi et al. 2007). On the other hand, double knockout mutant of MGD2/3, *mgd2mgd3*, showed comparable amount of galactolipids to WT in shoots under Pi-sufficient conditions (Kobayashi et al. 2009a, b). Type-B MGDs are localized on the outer envelope membrane of plastids and function together with DGD2; the MGD2/3-DGD2 pathway contributes to galactolipid synthesis in non-photosynthetic tissues such as roots and flowers (Awai et al. 2001; Nakamura et al. 2009a). Furthermore, the MGD2/3-DGD2 pathway is highly induced under Pi deficiency and is involved in membrane lipid remodeling under such conditions. Lipid analyses on *mgd2mgd3* indicated that type-B MGDs contribute ~ 50% of DGDG increase respond to Pi-starvation in shoots. In contrast, DGDG accumulation in roots respond to Pi-starvation is almost entirely dependent on MGD2/3-DGD2 pathway (Kobayashi et al. 2009a, b). Although the metabolic pathways of galactolipids in *Arabidopsis* have been investigated in detail (Shimojima and Ohta 2011), the regulation of galactolipid synthesis under acidic conditions remains unknown. In this study, we report that membrane lipid composition is altered under acidic conditions with or without Pi, and membrane lipid remodeling is possibly involved in the stress adaptation to acidic Pi deficiency.

## Materials and methods

### Plant materials and growth conditions

WT *Arabidopsis thaliana* was of the Columbia ecotype (Col-0). The double-knockout mutant of type-B MGDG synthases, *mgd2mgd3*, was obtained as described (Kobayashi et al. 2009a, b). Sterilized seeds were plated on half-strength Murashige and Skoog (MS) medium (Murashige and Skoog 1962) containing 1% (w/v) sucrose, 20 mM MES-KOH at pH 6.0 and 0.8% (w/v) agar. Seeds were incubated at 4 °C for 4 days in the dark and germinated under continuous white light (~ 50 µE m^−2^ s^−1^) at 23 °C for 10 days. Then, plants were transferred to three-fold diluted MGRL medium (1/3 MGRL) at pH ~ 6.0 (Fujiwara et al. 1992) for 5 days. For the stress treatments, plants were subsequently transferred to 1/3 MGRL medium, which was adjusted to pH 3.7 by using H_2_SO_4_ or HCl, and grown for another 7–9 days. The pH of the acidic medium was monitored and adjusted every day. For the control conditions, plants were transferred to 1/3 MGRL medium without adjusting the pH (initially at pH ~ 6.0). For Pi depletion, 1/3 MGRL medium without phosphate containing 10 mM MES-KOH at pH 6.0 was used for plant hydroponic culture as described above, whereas the pH was adjusted to 4.5 or 3.7 by using H_2_SO_4_ for the acidic Pi-deficient conditions.

### Estimation of ion concentrations in plant growth media

Ion concentrations in 1/3 MGRL medium with or without phosphate at pH 6.0 and 3.5 were calculated using GEOCHEM-EZ (Shaff et al. 2010). Parameters were set as follows: “Convergence criterion” was set at 1e–4; “Number of Interactions” was set at 50; “Solids” was set at “can precipitate”; “Fixed Ionic Strength” was set at 0.10.

### Lipid analysis

Lipid analyses were done essentially as described (Kobayashi et al. 2007). Total lipids were extracted from 22-days-old plant samples as described (Bligh and Dyer 1959). Polar lipids were separated by two-dimensional TLC on silica gel TLC plates (TLC Silica gel 60, Glass plates 20 × 20 cm; Merck) using the following solvent systems: chloroform/methanol/7 M ammonia (115:80:8, v/v/v) for the first dimension and chloroform/methanol/acetic acid/water (170:25:15:3, v/v/v/v) for the second dimension. Separated lipids were derivatized to fatty acid methyl esters and quantified by gas chromatography using pentadecanoic acid as an internal standard. Preparation of oligogalactolipids was performed as described (Barnes et al. 2016). Polar lipids were separated by TLC using the following solvent system: chloroform/methanol/acetic acid/water (85:20:10:4, v/v/v/v). Carbohydrates in the head group of lipids were visualized by spraying with a solvent (2.5 mM anthrone, 1.3 mM thiourea, 66% (v/v) sulfuric acid) and baking at 110 °C for 10 min. For the positive control of this analysis, an SFR2 assay in whole-plant tissue was done as described (Barnes et al. 2016). Briefly, a 14-days-old WT *Arabidopsis* rosette was floated on the assay buffer (20 mM acetic acid, 10 mM MgCl_2_, adjusted to pH 4.0 with K_2_HPO_4_) overnight under dim light.

### qPCR

Total RNA was extracted from 22-days-old plant samples using the SV Total RNA Isolation System (Promega). Reverse transcription was performed with 325 ng RNA using the PrimeScript RT reagent kit (Takara). qPCR was carried out using the SYBR Premix Ex Taq II (Takara) based on the manufacturer’s protocols, and signals were detected with the Thermal Cycler Dice Real Time System (Takara). The 2^−∆∆Ct^ value was calculated based on the expression of *UBQ10* as an internal standard. Sequences of primers are listed (Table SIII) as described (Bari et al. 2006; Murakawa et al. 2014).

### Isolation of microsomal membrane fraction

The microsomal membrane fraction was obtained essentially as described (Nakamura et al. 2009b; Murakawa et al. 2014). Shoots from 22-day-old plants were homogenized in the buffer (50 mM HEPES–KOH at pH 7.8, 2 mM EDTA, 1 mM MgCl_2_, 1 mM MnCl_2_), and tissue debris was removed by centrifugation at 300×*g*. To remove thylakoid membranes, the crude extract was centrifuged twice at 3000×*g* for 10 min, and the resulting supernatant was centrifuged at 125,000×*g* for 60 min to separate the microsomal fraction as a pellet from the soluble fraction. The microsomal fraction was suspended in 50 mM HEPES–KOH at pH 7.8, and polar lipids were extracted from the microsomal fraction by addition of 10 vol of chloroform/methanol (2:1, v/v).

### Measurement of Pi content

Pi was extracted from 22-days-old plant samples and quantified by phosphomolybdate colorimetric assay as described (Chiou et al. 2006). Briefly, samples were homogenized with extraction buffer (10 mM Tris, 1.0 mM EDTA, 100 mM NaCl, 1.0 mM β-mercaptoethanol, pH 8.0) and centrifuged at 12,000×*g* for 10 min. A portion of the resulting supernatant (100 μL) was mixed with 900 μL of 1% (v/v) glacial acetic acid and incubated at 42 °C for 30 min. The mixture was then centrifuged at 12,000×*g* for 5 min. A portion of the resulting supernatant (300 μL) was mixed with 700 μL of assay solution (0.35% [w/v] NH_4_MoO_4_, 0.43 M H_2_SO_4_, 1.4% [w/v] ascorbic acid) and incubated at 42 °C for 30 min. The Pi content was measured at *A*_820_ based on the calibration curve obtained from the Pi standard solutions.

### Liposome preparation

Total lipids were extracted from spinach as described (Bligh and Dyer 1959) and separated by TLC using a solvent system consisting of acetone/toluene/water (136:45:12, v/v/v). Spinach DGDG and PC were eluted from the silica gel by using chloroform/methanol (1:1, v/v) and methanol, respectively. To form liposomes, 2 mg of DGDG and/or PC was mixed with 2 mg of lecithin, which was prepared by removing proteins and triacylglycerol from Asolectin from soybean (Sigma) with chloroform/iced acetone (1:6, v/v). Each mixture of lipid suspension was dried under nitrogen gas in a glass test tube and re-hydrated with 300 µL of buffer solution (2 mM pyranine, 5 mM KH_2_PO_4_, 100 mM KCl, 1 nmol valinomycin/mg·lipid, adjusted to pH 7.0 with KOH) at 37 °C for 10 min. Then, a liposome suspension was prepared by sonication using an ultrasonic cleaner (Branson 3210, Yamato) for 20 min and an ultrasonic disruptor (UD-201, TOMY: output, 1; duty, 30) for 60 s. To remove disordered liposomes, each suspension was centrifuged at 3000 ×* g* for 5 min. Then, each liposome suspension was dialyzed against pyranine-free buffer (5 mM KH_2_PO_4_, 100 mM KCl, adjusted to pH 7.0 with KOH) to remove pyranine present outside of the liposomes.

### Measurement of proton permeability of liposomes

The measurement of proton permeability of the liposomes was conducted as described (Shimada et al. 2008), with some modifications. The internal pH of the liposomes was estimated based on the fluorescence excitation spectrum of pyranine (Kano and Fendler 1978). Each liposome suspension (100 μL) was mixed with 1.9 mL of acidic buffer (5 mM KH_2_PO_4_, 100 mM KCl, adjusted to pH 4.5 with HCl), and the excitation spectrum of fluorescence emission at 510 nm was measured for 30 min with a fluorescence spectrophotometer (F-2700, HITACHI). For the intensity at 0 min, 100 µL of liposome suspension was mixed with 1.9 mL of neutral buffer (5 mM KH_2_PO_4_, 100 mM KCl, adjusted to pH 7.0 with KOH). The internal pH of the liposomes was calculated based on the logarithm of the ratio of emission intensities with excitation at 400 and 460 nm: log(*I*_400 nm_/*I*_460 nm_). To obtain the calibration curve in Figure S1, log(*I*_400 nm_/*I*_460 nm_) of standard solutions (33 µM pyranine; 5 mM KH_2_PO_4_; 100 mM KCl; at pH 4.5, 5.0, 5.5, 6.0, 6.5, 7.0 and 7.5) was plotted against the solution pH.

### Measurement of chlorophyll content

Total chlorophyll was extracted in 80% (v/v) acetone at 4 °C. Samples were centrifuged at 12,000 ×* g* for 5 min, and the supernatant was used for the measurement of the absorbance with a spectrophotometer (UV-1850, Shimadzu). Total chlorophyll was calculated using the following formula (Porra 2002): Total chlorophyll (nmol·mL^−1^) = 19.54 × (*A*_646.8 _− *A*_720_) + 8.29 × (*A*_663.2 _− *A*_720_).

## Results

### Lipid metabolism in WT *Arabidopsis* grown under acidic conditions

**Fig. 1:**
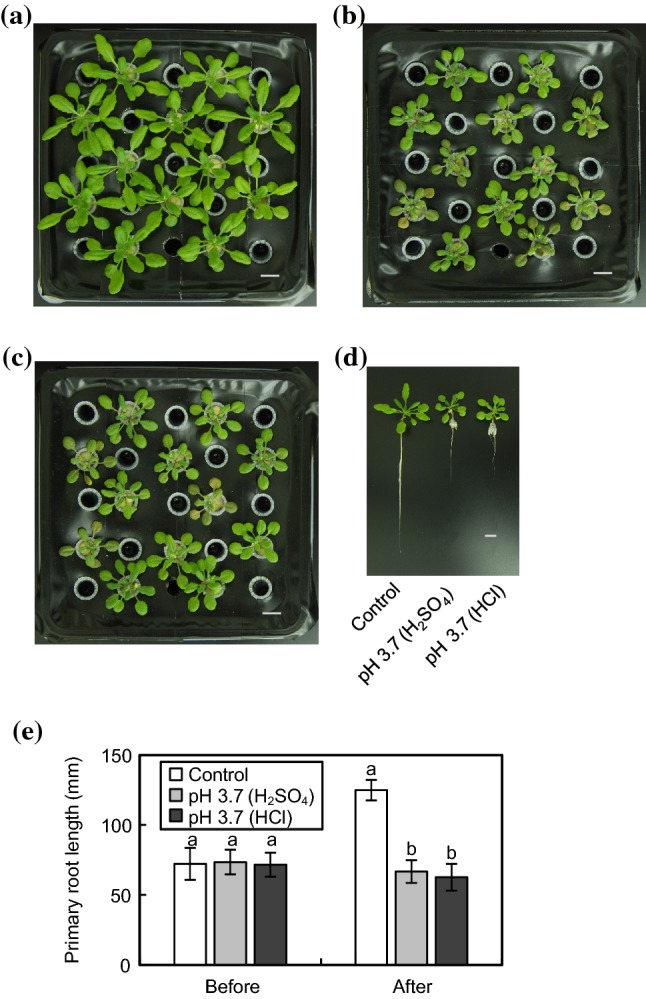
Growth phenotypes of WT *Arabidopsis* plants under acidic conditions. Plant seedlings were germinated on 1/2 MS medium for 10 days and then transferred to 1/3 MGRL medium for 5 days. Acidic stress treatments were subsequently conducted; Plants were grown in 1/3 MGRL medium as the control (**a**) or acidic 1/3 MGRL medium at pH 3.7 adjusted with H_2_SO_4_ (**b**) or HCl (**c**) for another 7 days. **d** Whole morphology of WT *Arabidopsis* grown under acidic conditions. Bars = 1 cm. **e** Primary root length of WT *Arabidopsis* before and after a week of acidic stress treatment. Values are the mean ± SD (*n* = 20). Different letters represent significant differences based on the Tukey–Kramer multiple comparison test (*p* < 0.05)

To assess the effects of acidic conditions on plant growth, we investigated morphological phenotypes of WT *Arabidopsis* grown at pH 3.7. To compare the effects of the counteranion of the acid, we adjusted the medium pH with H_2_SO_4_ or HCl. SO_4_^2−^ is not effective at permeating membranes, whereas Cl^−^ is highly permeating (Hinsinger et al. 2003). As compared with plants grown under control conditions, rosette leaf expansion and primary root elongation were arrested at pH 3.7 (Fig. 1). As for the comparison between H_2_SO_4_ and HCl, there were no obvious differences in either the shoot or root phenotype, showing that the protons released from the acids, but not the counteranions, were responsible for growth impairment under acidic conditions. To clarify whether the increase in the proton concentration would cause changes in ion balances in the growth medium, we calculated the ion concentrations in our growth medium at pH 6.0 and 3.5 with the chemical speciation program GEOCHEM-EZ (Shaff et al. 2010). Based on this simulation, insoluble salts that prevent plants absorbing nutrients are not formed either at pH 6.0 or pH 3.5 (Table SI), indicating that plants do not suffer from nutritional deprivation under acidic conditions in our experimental protocol.

**Fig. 2:**
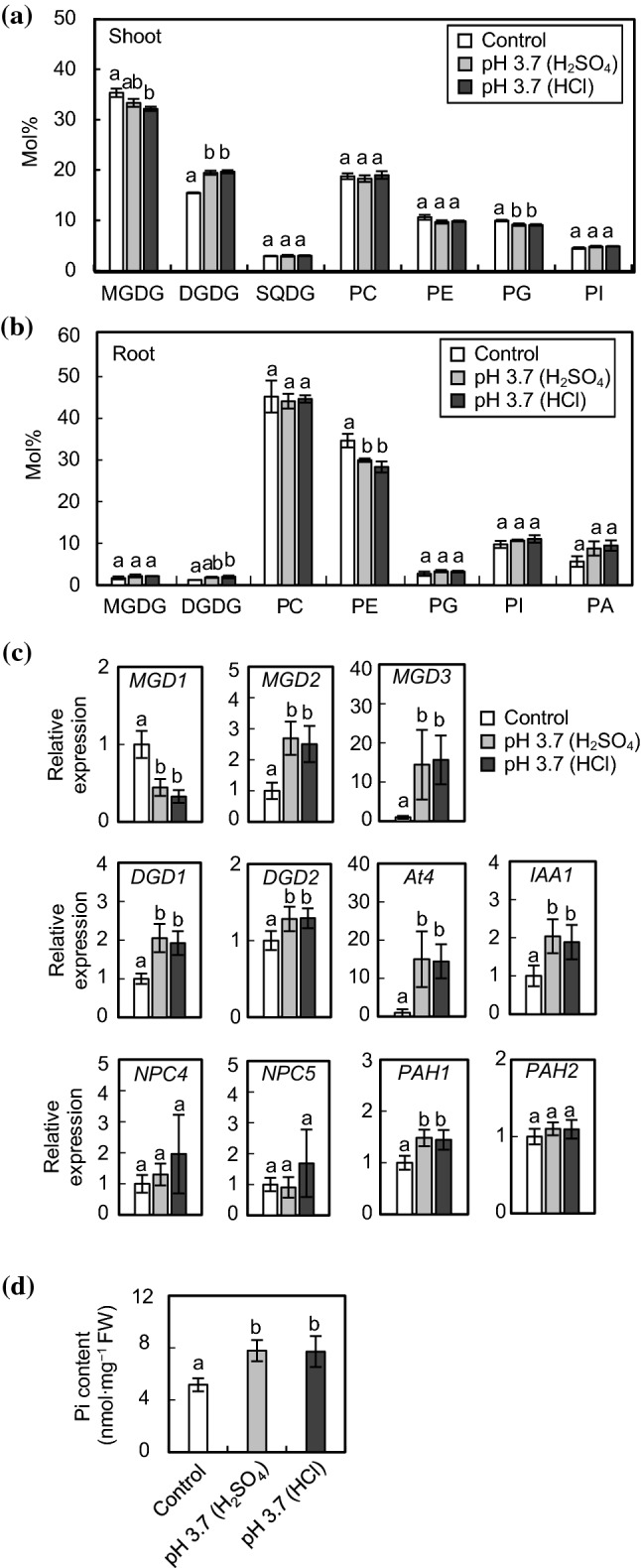
Lipid, gene expression and Pi content analyses of WT *Arabidopsis* plants grown under acidic conditions. Seedlings were germinated and grown as in Fig. 1. Composition of polar membrane lipids in shoots (**a**) and roots (**b**). SQDG, sulfoquinovosyldiacylglycerol; PI, phosphatidylinositol; PA, phosphatidic acid. Values are the mean ± SD (*n* = 3). **c** Gene expression analyses in rosette leaves. Relative mRNA abundance of each gene in expanded rosette leaves was analyzed by qPCR using *UBQ10* as an internal control. Values are the mean ± SD (*n* = 8) of expression fold differences relative to that of control conditions. **d** Pi content in rosette leaves. Values are the mean ± SD (*n* = 4). Different letters represent significant differences based on the Tukey–Kramer multiple comparison test (*p* < 0.05)

To examine whether membrane lipid composition is altered in response to acidic stress, lipid analyses were conducted with shoots and roots of WT *Arabidopsis* grown at pH 3.7, which was adjusted with H_2_SO_4_ or HCl (Fig. 2). Similar to the morphological phenotypes, there were no significant differences in lipid composition between the two kinds of acidic stresses, H_2_SO_4_ and HCl. In shoots of WT plants grown under acidic conditions, the molar percentage of DGDG was significantly increased by ~ 25% as compared with plants grown under control conditions (Fig. 2a). Meanwhile, phosphatidylcholine (PC) and phosphatidylethanolamine (PE), which are considerably degraded under Pi starvation, were not decreased under acidic conditions. Instead, MGDG and phosphatidylglycerol (PG) were slightly decreased. In roots, no marked change in molar percentages of galactolipids in response to acidic stress was observed (Fig. 2b). Fatty acid composition of lipids in shoots and roots of WT under acidic conditions is shown in Figs. S1 and S2, respectively. There were no marked differences in fatty acid composition of MGDG and DGDG under acidic conditions, suggesting that the contribution of MGD1, MGD2 and MGD3 to MGDG and DGDG synthesis under acidic conditions seems to be comparable between control and acidic conditions.

We also examined transcriptional changes in leaves from WT plants grown under acidic conditions by qPCR (Fig. 2c). With respect to MGDG synthase genes, *MGD1* expression was significantly downregulated, whereas *MGD2* and *MGD3* were significantly upregulated. As for DGDG synthase genes, expression of both *DGD1* and *DGD2* was increased, but *DGD1* was more notably increased by ~ twofold. Because *MGD2/3* and *DGD1/2* are inducible under Pi starvation (Awai et al. 2001; Kelly and Dörmann 2002; Kelly et al. 2003), we also analyzed the expression of *At4*, which is one of the Pi starvation marker genes but is not involved in lipid remodeling (Martín et al. 2000). *At4* transcripts were significantly increased under acidic conditions, suggesting that a part of the Pi starvation response is induced at the transcriptional level by acidic stress. In addition, the expression of an auxin-responsive gene, *IAA1*, was also upregulated, consistent with the previous evidence that acidic stress induces auxin accumulation and auxin response signaling (Lager et al. 2010; Hachiya et al. 2014). NPC4/5 and PAH1/2 are involved in phospholipid degradation and supply substrates for galactolipid synthesis under Pi-deficient conditions (Nakamura et al. 2005; Gaude et al. 2008; Nakamura et al. 2009b). Although *NPC4/5* and *PAH1* are Pi starvation–responsive genes, only *PAH1* expression was increased under acidic conditions, suggesting that acidic stress does not activate all aspects of Pi starvation–inducible phospholipid degradation. These results are consistent with the lipid analysis (Fig. 2a). There were no significant differences in gene expression patterns between medium containing H_2_SO_4_ and HCl, in agreement with our results for plant growth phenotypes and lipid composition.

To confirm whether or not plants had sufficient Pi under acidic conditions, the Pi content in leaves was measured (Fig. 2d). The results showed that Pi concentration was not decreased, rather increased, at pH 3.7, indicating that plants were not Pi starved during acidic stress with either H_2_SO_4_ or HCl.

### Lipid metabolism in *mgd2mgd3 * Arabidopsis grown under acidic conditions

**Fig. 3:**
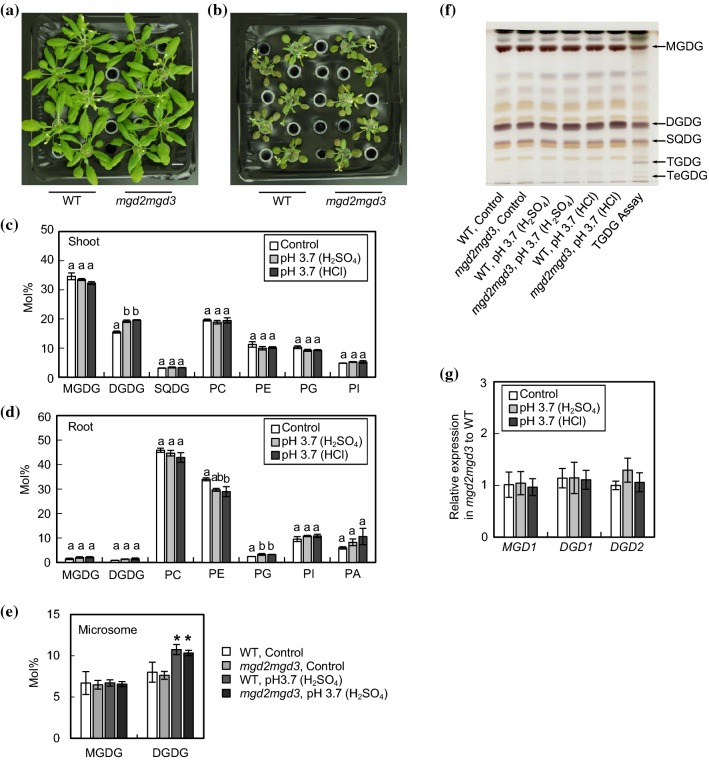
Lipid analyses of *mgd2mgd3* plants grown under acidic conditions. Seedlings were germinated and grown as in Fig. 1. Comparison of growth phenotypes between WT and *mgd2mgd3* after 9 d of acidic stress treatments at the control (**a**) or pH 3.7 with H_2_SO_4_ (**b**). Bars = 1 cm. Composition of polar membrane lipids of *mgd2mgd3* in shoots (**c**) and roots (**d**). Values are the mean ± SD (*n* = 3). Different letters represent significant differences based on the Tukey–Kramer multiple comparison test (*p* < 0.05). **e** Galactolipid content in shoot microsomal membrane fractions of WT and *mgd2mgd3* plants. Values are the mean ± SD (*n* = 3). Asterisks represent significant differences from the control growth conditions (*p* < 0.05, Student’s *t* test). **f** Glycolipid staining on a thin-layer chromatogram. Sugars in head groups of lipids are visualized by the anthrone-sulfuric acid assay. As the positive control for oligogalactolipid, a 14-day-old WT *Arabidopsis* rosette was floated on the assay buffer (20 mM acetic acid, 10 mM MgCl_2_, pH adjusted to 4.0 with K_2_HPO_4_) overnight (Barnes et al. 2016). **g** Relative expression of *MGD1*, *DGD1* and *DGD2* in rosette leaves of *mgd2mgd3* to WT. Relative mRNA abundance of each gene in expanded rosette leaves was analyzed by qPCR using *UBQ10* as an internal control. Values are the mean ± SD (*n* = 6) of expression fold differences relative to that of the same conditions in WT

For further investigation of lipid metabolism during acidic stress, we grew *mgd2mgd3*, the double knockout mutant of type-B MGD genes, under acidic conditions. Defects in *MGD2/3* did not lead to obvious changes in growth phenotypes under acidic conditions, compared to WT (Fig. 3a, b). As for lipid compositions, *mgd2mgd3* also showed the same pattern as WT (Figs. 2a, b, 3c, d). These results clearly indicated that, unlike Pi-depleted conditions, MGD2 and MGD3 were not involved in the DGDG accumulation but MGD1-DGD1 pathway was responsible for DGDG accumulation in shoots under acidic conditions. To further examine the accumulation site of DGDG in shoots under acidic conditions, microsomal membranes were obtained from shoots of WT and *mgd2mgd3* grown at pH 3.7 adjusted with H_2_SO_4_, and their galactolipid content was analyzed (Fig. 3e). The microsomal membranes obtained by differential centrifugation mainly consist of the plasma membrane and membranes derived from the endoplasmic reticulum, Golgi apparatus, trans-Golgi network, tonoplast, mitochondria and the envelope membranes of chloroplasts. Thus, the overall amount of MGDG and DGDG, which are the main constituents of thylakoid membrane, was decreased in the microsomal membranes as compared with the total membrane lipids (Figs. 2a, 3c, e). However, the amounts of DGDG in microsomal membranes of both WT and *mgd2mgd3* grown under acidic conditions were significantly increased by ~ 35% as compared with that of control conditions, whereas MGDG showed no increase. These results suggest that, under acidic stress, DGDGs were accumulated in the chloroplast envelope membranes or the extraplastidic membranes similarly to Pi starvation.

In addition to the two MGD-DGD pathways (MGD1-DGD1 pathway and MGD2/3-DGD2 pathway) plants have an alternative galactolipid synthetic mechanism, galactolipid:galactolipid galactosyltransferase (GGGT), using MGDG as the donor of a galactose moiety (Dörmann and Benning 2002). In *Arabidopsis*, it has been suggested that cytosolic acidification and Mg^2+^ leakage from chloroplasts caused by freezing stress triggers GGGT activation and synthesis of oligogalactolipids, mainly trigalactosyldiacylglycerol (TGDG) and tetragalactosyldiacylglycerol (TeGDG) (Moellering et al. 2010; Barnes et al. 2016). Thus, we analyzed oligogalactolipids from WT and *mgd2mgd3* grown under acidic conditions (Fig. 3f). MGDG, DGDG and sulfoquinovosyldiacylglycerol (SQDG) were detected, yet neither TGDG nor TeGDG were visible, indicating that oligogalactolipids that contain three or more sugar units did not accumulate in *Arabidopsis* membranes under acidic conditions, indicating that TGDG synthesis catalyzed by GGGT is not activated under acidic conditions.

We also examined transcriptional changes in leaves from *mgd2mgd3* plants grown under acidic conditions by qPCR (Fig. 3g). Expression of *MGD1*, *DGD1* and *DGD2* in *mgd2mgd3* was similar to those in WT both under control and acidic conditions, suggesting that an increase in DGDG under acidic conditions in *mgd2mgd3* is not due to the upregulation of these gene expression compared with WT.

### Lipid metabolism in WT *Arabidopsis* grown under acidic Pi deficiency

**Fig. 4:**
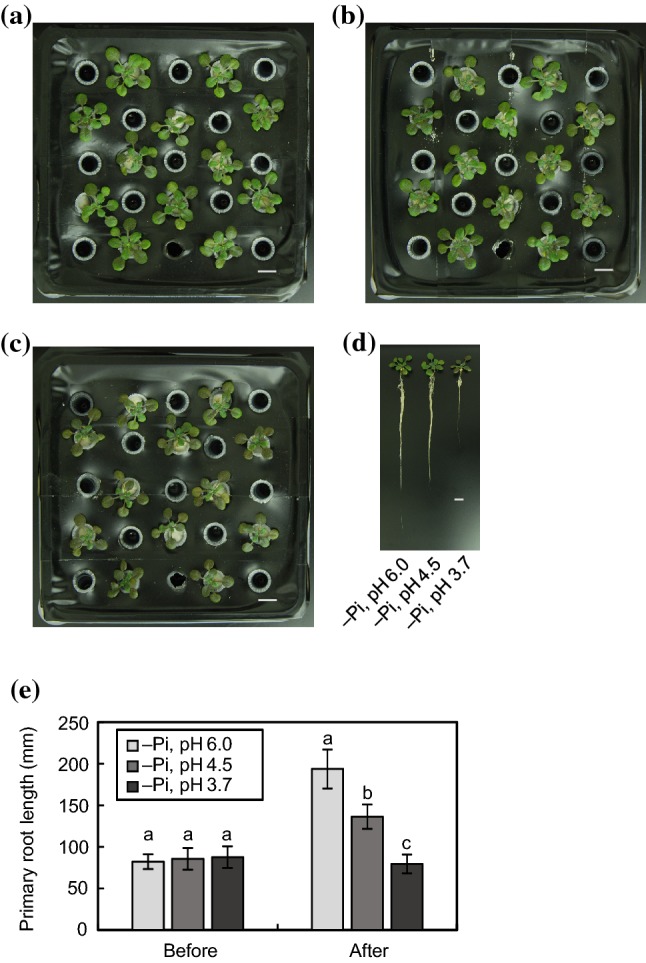
Growth phenotypes of WT *Arabidopsis* under acidic Pi-deficient conditions. Plant seedlings were germinated on 1/2 MS medium for 10 days and transferred to 1/3 MGRL medium without phosphate (–Pi) containing 10 mM MES-KOH (pH 6.0) for 5 days. Then, acidic Pi depletion stress treatments were subsequently conducted. Plants were grown in 1/3 MGRL medium –Pi at pH 6.0 (**a**), 4.5 (**b**) or 3.7 (**c**) adjusted with MES-KOH (pH 6.0) or H_2_SO_4_ (pH 4.5 and 3.7) for another 7 days. **d** Whole morphology of WT *Arabidopsis* grown under acidic Pi-deficient conditions. Bars = 1 cm. **e** Primary root length of WT *Arabidopsis* before (–Pi, 5 days) and after (–Pi, 12 days) a week of acidic Pi depletion stress treatments. Values are the mean ± SD (*n* = 20). Different letters represent significant differences based on the Tukey–Kramer multiple comparison test (*p* < 0.05)

As acidic stress induced the expression of some Pi starvation-responsive genes (Fig. 2c), we further examined how a combined stress of an acidic environment under Pi deficiency affects the physiology of *Arabidopsis*. We confirmed that a change in pH from 6.0 to 3.5 does not cause the formation of insoluble salts in Pi-depleted growth medium (Table SII). Then, we observed the morphological phenotypes of WT *Arabidopsis* grown under Pi-deficient conditions at pH 6.0, 4.5 or 3.7 (Fig. 4). The size of rosette leaves was comparable among the three pH conditions under Pi deficiency, whereas the primary root growth was repressed with a decrease in pH (Fig. 4d, e), suggesting that plants have the ability to modify their root growth depending on external pH conditions even under Pi deficiency.

**Fig. 5:**
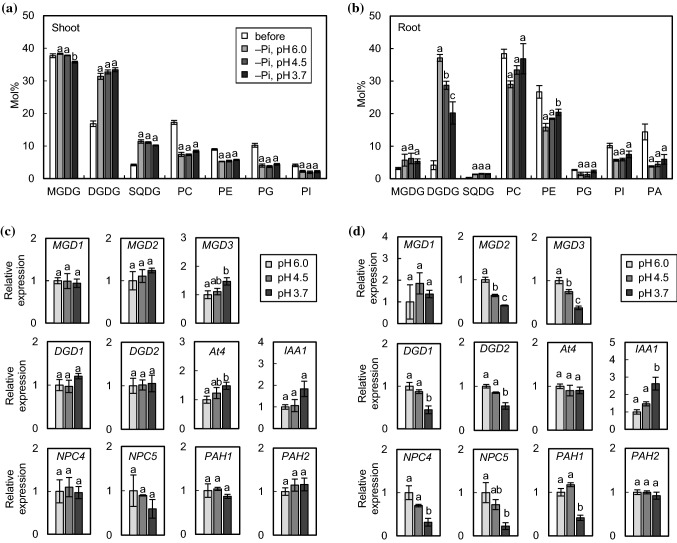
Lipid and gene expression analyses of WT *Arabidopsis* grown under acidic Pi-deficient conditions. Seedlings were germinated and grown as in Fig. 4. Lipid composition of polar membrane lipids in shoots (**a**) and roots (**b**) was measured before (–Pi, 5 days) and after (–Pi, 12 days) a week of acidic Pi depletion stress treatments. Values are the mean ± SD (*n* = 3). Gene expression analyses in rosette leaves (**c**) and roots (**d**) of WT *Arabidopsis* grown under acidic Pi-deficient conditions. Relative mRNA abundance of each gene was analyzed by qPCR with *UBQ10* as an internal control. Values are the mean ± SD (*n* = 3) of expression fold differences from that of pH 6.0. Different letters represent significant differences based on the Tukey–Kramer multiple comparison test (*p* < 0.05)

We subsequently conducted lipid analyses of WT *Arabidopsis* grown under acidic Pi-deficient conditions (Fig. 5a, b). We confirmed Pi starvation-induced lipid remodeling in both shoots and roots when compared the Pi-starved seedlings for 5 days (before) with those for 12 days (–Pi, pH 6.0) as shown in Fig. 5a and b. In shoots, the effect of acidic stress under Pi deficiency was not remarkable and the lipid compositions of seedlings under three different pH conditions (–Pi, pH 6.0, pH 4.5 and pH 3.7) were comparable (Fig. 5a). In roots, by contrast, the amount of DGDG at a lower pH (pH 4.5 and pH 3.7) was significantly lower than that at pH 6.0 (Fig. 5b), indicating that the Pi starvation-induced lipid remodeling in roots was partially repressed by acidic stress. Fatty acid composition of galactolipids in shoots and roots under acidic Pi deficiency showed no obvious change (Figs. S3, S4).

We also performed qPCR analyses in rosette leaves and roots from WT *Arabidopsis* grown under acidic Pi-deficient conditions (Fig. 5c, d). It should be noted that the base expression level, at pH 6.0, of Pi starvation–responsive genes, especially *MGD2/3*, *DGD1/2*, *At4*, *NPC4/5* and *PAH1*, represents the transcriptional level already induced by Pi starvation as compared with Pi-sufficient conditions. In rosette leaves, *MGD3* and *At4* were upregulated under Pi-deficient conditions at pH 3.7 as compared with pH 6.0, although the other genes were not significantly changed (Fig. 5c). Meanwhile, in roots, *MGD2/3*, *DGD1/2*, *NPC4/5* and *PAH1* were downregulated under acidic Pi-deficient conditions, indicating that lipid remodeling during Pi depletion in roots is transcriptionally repressed by acidic stress (Fig. 5d). *At4* and *IAA1* were not downregulated in roots under acidic Pi-deficient conditions. Thus, although the effect of acidic stress to the expression of Pi starvation-induced genes might be very limited, it was clearly shown that acidic stress represses expression of genes involved in Pi starvation-induced lipid remodeling in roots and thus represses the lipid remodeling.

### Membrane lipid remodeling in roots under acidic Pi deficiency

**Fig. 6:**
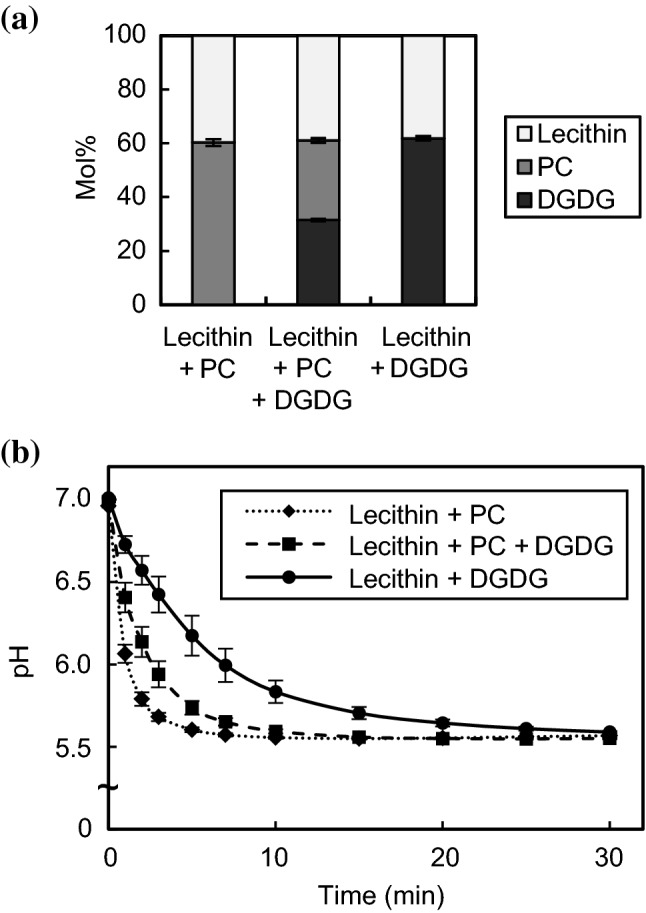
Proton permeability of membranes comprising DGDG and PC from spinach. **a** Lipid composition of liposomes. **b** Time course of changes in internal pH of liposomes. The fluorescence emission at 510 nm of the pH-sensitive probe pyranine present within the liposome was measured at 1, 2, 3, 5, 7, 10, 15, 20, 25 and 30 min after 100 µL of liposome suspension (pH 7.0) was mixed with 1.9 mL of acidic buffer (pH 4.5). For the intensity at 0 min, the liposome suspension was diluted with neutral buffer (pH 7.0). Internal pH was calculated based on the calibration curve showing the relationship between solution pH and the logarithm of the ratio of the emission intensity with excitation at 400 to that at 460 nm (Figure S5). Values are the mean ± SD (*n* = 3)

Pi starvation-induced lipid remodeling in roots was partially repressed by acidic stress, but still DGDG content in Pi-starved roots at lower pH was higher than that of Pi-sufficient conditions (Figs. 3d, 5b). Previous research has suggested that membranes comprising glycolipids are resistant to proton permeation relative to phospholipids (Fuks and Homblé 1996; Paula et al. 1996). The proton-resisting characteristics of glycolipids are thought to result from the abundance of hydroxyl groups in the sugar moieties (Róg et al. 2007; Shimada et al. 2008; Wang et al. 2012). To compare the proton permeability of membranes comprising DGDG and PC from plants, we prepared liposomes including DGDG and/or PC extracted from spinach at three ratios (Fig. 6a). Proton permeability of these membranes was evaluated by observation of changes in the internal pH of liposomes after dilution of the liposome suspension (pH 7.0) with acidic buffer (pH 4.5) (Fig. 6b). Notably, the proton influx for the liposome membrane made of lecithin, PC and DGDG (~ 30% DGDG) was lower than that made of lecithin and PC (0% DGDG), and was higher than that made of lecithin and DGDG (~ 60% DGDG). These results clearly indicated that a membrane comprising a plant DGDG has a lower proton permeability than that of PC in vitro.

**Fig. 7:**
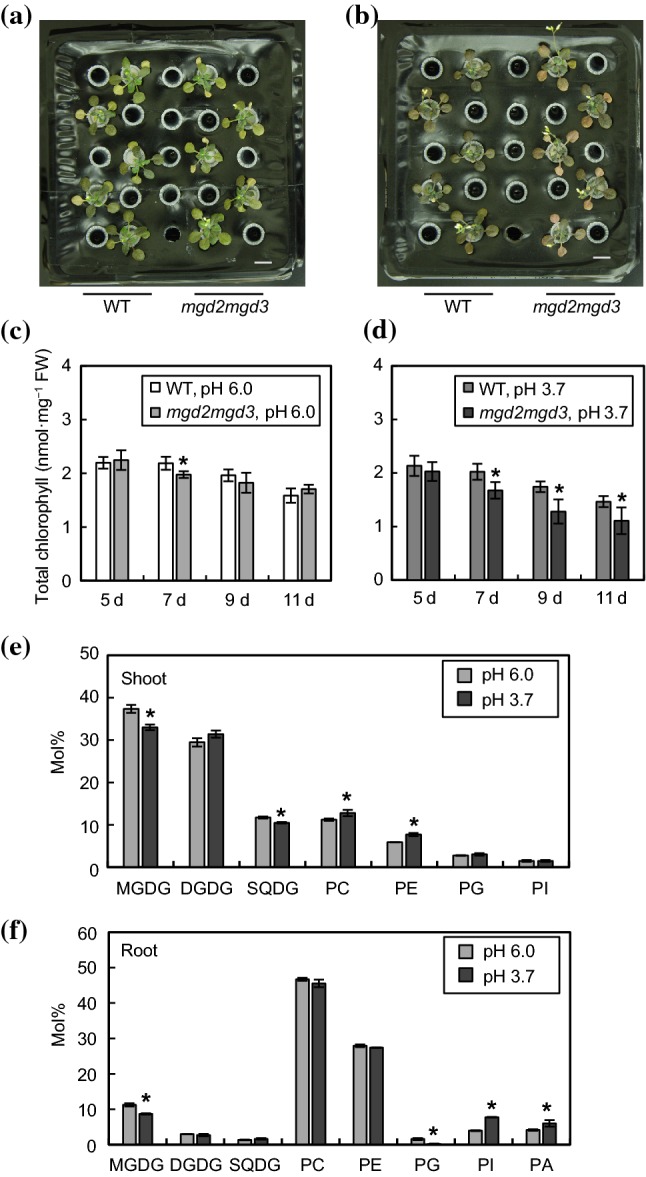
Comparison of phenotypes of WT *Arabidopsis* and *mgd2mgd3* under acidic Pi-deficient conditions. Seedlings were germinated and grown as in Fig. 4. Comparison of growth phenotypes between WT and *mgd2mgd3* after 9 days of acidic Pi depletion stress treatments at pH 6.0 (**a**) or 3.7 (**b**). Bars = 1 cm. Chlorophyll content in rosettes from WT and *mgd2mgd3* after 5, 7, 9 and 11 days of acidic Pi depletion stress treatments at pH 6.0 (**c**) or 3.7 (**d**). Values are the mean ± SD (*n* = 4). Asterisks represent significant differences between WT and *mgd2mgd3* for each time point (*p* < 0.05, Student’s *t*-test). Lipid composition of polar membrane lipids in shoots (**e**) and roots (**f**) of *mgd2mgd3* was measured after 7 days of acidic Pi depletion stress treatments at pH 6.0 or 3.7. Values are the mean ± SD (*n* = 3). Asterisks represent significant differences between pH 6.0 and pH 3.7 for each lipid (*p* < 0.05, Student’s *t*-test)

To investigate the physiological effects of DGDG accumulation in root membranes during acidic Pi starvation stress, we compared the growth of WT *Arabidopsis* with that of *mgd2mgd3* because DGDG scarcely increases in roots of *mgd2mgd3* during Pi depletion (Kobayashi et al. 2009a, b). Although there were no obvious phenotypic differences between WT and *mgd2mgd3* at pH 6.0 (Fig. 7a), *mgd2mgd3* showed notably discolored rosette leaves at pH 3.7, whereas WT did not (Fig. 7b). We also quantified the chlorophyll content in rosettes from WT and *mgd2mgd3*. At pH 6.0, the chlorophyll content in both WT and *mgd2mgd3* was similarly decreased during the time course (Fig. 7c). In contrast, at pH 3.7, *mgd2mgd3* showed a more rapid decrease in chlorophyll content than WT (Fig. 7d), suggesting that *mgd2mgd3* is more sensitive to acidic Pi deficiency than WT. We also analyzed the lipid composition of *mgd2mgd3* plants grown under acidic Pi-deficient conditions (Fig. 7e, f). In shoots and roots of *mgd2mgd3*, the effect of acidic stress under Pi deficiency was not remarkable compared with the case of WT (Fig. 5a, b) and the lipid compositions of seedlings under two different pH conditions (–Pi, pH 6.0 and pH 3.7) were not remarkably different (Fig. 7e, f). However, when compared with WT, the amount of DGDG in roots of *mgd2mgd3* was markedly lower than that of WT (Figs. 5b, 7f).

These results indicate that the function of type-B MGDG synthases and, thus, the DGDG increase triggered by Pi starvation, especially in roots, contributes to the acquisition of whole-plant stress tolerance under acidic Pi-deficient conditions.

## Discussion

DGDG in shoots from both WT and *mgd2mgd3* grown at pH 3.7 showed an ~ 25% increase in molar percentages as compared with plants grown under control conditions (Figs. 2a, 3c). From previous research, an ~ 25% increase in DGDG can lead to stress tolerance in plants. For example, high temperature induces a DGDG increase of 23%, and a partial defect in DGD1 in *Arabidopsis* reduces thermotolerance (Chen et al. 2006). DGDG is also increased by 28% in *Arabidopsis* during drought stress (Gigon et al. 2004). Moreover, transgenic tobacco (*Nicotiana tabacum*) lines that overexpress the rice MGD gene have a 11–24% higher DGDG content as compared with WT and show enhanced salt tolerance (Wang et al. 2014). Although the mechanisms remain unclear, these results together suggest that an increase of only ~ 25% in DGDG possibly mediated by *DGD1* should be sufficient to achieve tolerance to various abiotic stresses. As the results of gene expression analyses in WT *Arabidopsis* grown under acidic conditions, transcriptional regulation of galactolipid metabolism during acidic stress resembled that of Pi starvation (Fig. 2c). One possibility is that auxin-cytokinin interaction is the key regulation mechanisms of galactolipid metabolism responding to acidic stress. Low-pH stress induces the accumulation of auxin and a reduction in cytokinins in *Arabidopsis* shoots (Hachiya et al. 2014). Auxin signaling is involved in activation of *MGD2/3* and lipid remodeling during Pi depletion (Kobayashi et al. 2006; Narise et al. 2010). Conversely, exogenous cytokinin treatments repress DGDG accumulation even under Pi deficiency, especially in root membrane (Kobayashi et al. 2006). In addition, *MGD1* is transcriptionally up-regulated via cytokinin signaling (Yamaryo et al. 2003; Shimojima and Ohta 2011). Thus, transcriptional upregulation of *MGD2/3* concomitant with downregulation of *MGD1* under acidic conditions might be achieved by an auxin-cytokinin interaction. Although both *MGD2* and *MGD3* were transcriptionally upregulated by acidic stress, *mgd2mgd3* showed a comparable increase of DGDG in shoots relative to WT under acidic conditions (Figs. 2a, 3c), indicating that MGD1-DGD1 pathway is responsible for DGDG accumulation in shoots under acidic conditions. The relative expression of *NPC4/5*, which supply diacylglycerol for the MGD2/3-DGD2 pathway during Pi depletion (Nakamura et al. 2005; Gaude et al. 2008), was comparable at pH 3.7 and control conditions, whereas that of *PAH1*, which supplies the substrate for the MGD1-DGD1 pathway (Nakamura et al. 2009b), was upregulated at pH 3.7 (Fig. 2c). Lack of enough substrate supply might be the cause of non-engagement of type-B MGDs in DGDG accumulation during acidic stress. Moreover, it should also be considered for clarifying the regulatory mechanism of MGDG synthesis under acidic conditions that MGD activity is regulated in a redox-dependent manner (Yamaryo et al. 2006), and that jasmonic acid biosynthesis and signaling were involved in MGDG steady state level by regulating MGD1 activity and *MGD3* expression (Chevalier et al. 2019).

Phospholipids in membrane are thought to constitute up to one-third of total Pi source in plant cell. Thus, replacing phospholipids with non-phosphorus lipids can be advantageous in growth enhancement during Pi depletion. We showed that DGDG increase in root membranes during Pi depletion is partially repressed at a lower pH while other Pi starvation responses, such as expression of *At4* and the auxin signaling component *IAA1*, are maintained (Fig. 5b, d). In addition, preferential root growth during Pi depletion was inhibited at a lower pH, in parallel with the repression of membrane lipid remodeling (Fig. 4e). The increase in surface area caused by root growth enhancement could be beneficial for taking up nutrients under Pi deficiency but also would be harmful in low-pH environments. The results suggest that plants optimize root growth by controlling the membrane lipid remodeling to adapt to the pleiotropic stress effects of acidic Pi deficiency.

Membrane lipid remodeling during Pi depletion has also been considered to play a role in Al^3+^ tolerance. Ca^2+^ usually stabilizes plasma membrane by binding to the negative charge on the membrane, mainly the phosphate group of phospholipids. Al^3+^, which is one of the most rhizotoxic ions in acid soils in nature, is thought to destabilize cell membranes by replacing membrane-bound Ca^2+^ (Kobayashi et al. 2013). Actually, *pah1pah2* double mutant includes more phospholipids in cell membranes than WT *Arabidopsis* (Nakamura et al. 2009b) and is more sensitive to Al^3+^ than WT during Pi depletion (Kobayashi et al. 2013). Moreover, induction of membrane lipid remodeling by Pi depletion enhances Al^3+^ tolerance in rice (Maejima and Watanabe 2014). Similar to Al^3+^, proton is also thought to destabilize cell membranes (Kobayashi et al. 2013). In this study, we showed that liposome membranes that include DGDG obtained from plants have characteristics of a proton barrier in vitro (Fig. 6b). In addition, the *mgd2mgd3* double mutant, in which membrane lipid remodeling in roots is deficient, showed impairment of adaptation to acidic Pi deficiency (Fig. 7), suggesting the advantage of galactolipid-enriched extraplastidic membranes in roots under acidic conditions. A greater understanding of the physicochemical and biological characteristics of membranes comprising galactolipid and phospholipid is required to clarify the physiological significance of membrane lipid alterations in plants grown under various complicated environmental stresses. Further analyses under various combined stress conditions will provide us new insights into stress adaptation mechanisms in plants.

## Electronic supplementary material

Below is the link to the electronic supplementary material.

Supplementary material 1 (PDF 483 kb)

Supplementary material 2 (DOCX 42 kb)

Supplementary material 3 (PDF 129 kb)
