# Supplementary material for: Lipid remodeling under acidic conditions and its interplay with low Pi stress in Arabidopsis
Source: Plant Mol Biol. 2019 Jun 14;101(1):81–93. doi: 10.1007/s11103-019-00891-1 (PMC6695348; doi:10.1007/s11103-019-00891-1)
Supplement: Supplementary file 1 — Supplementary material 1 (PDF 483 kb) [file 11103_2019_891_MOESM1_ESM.pdf]

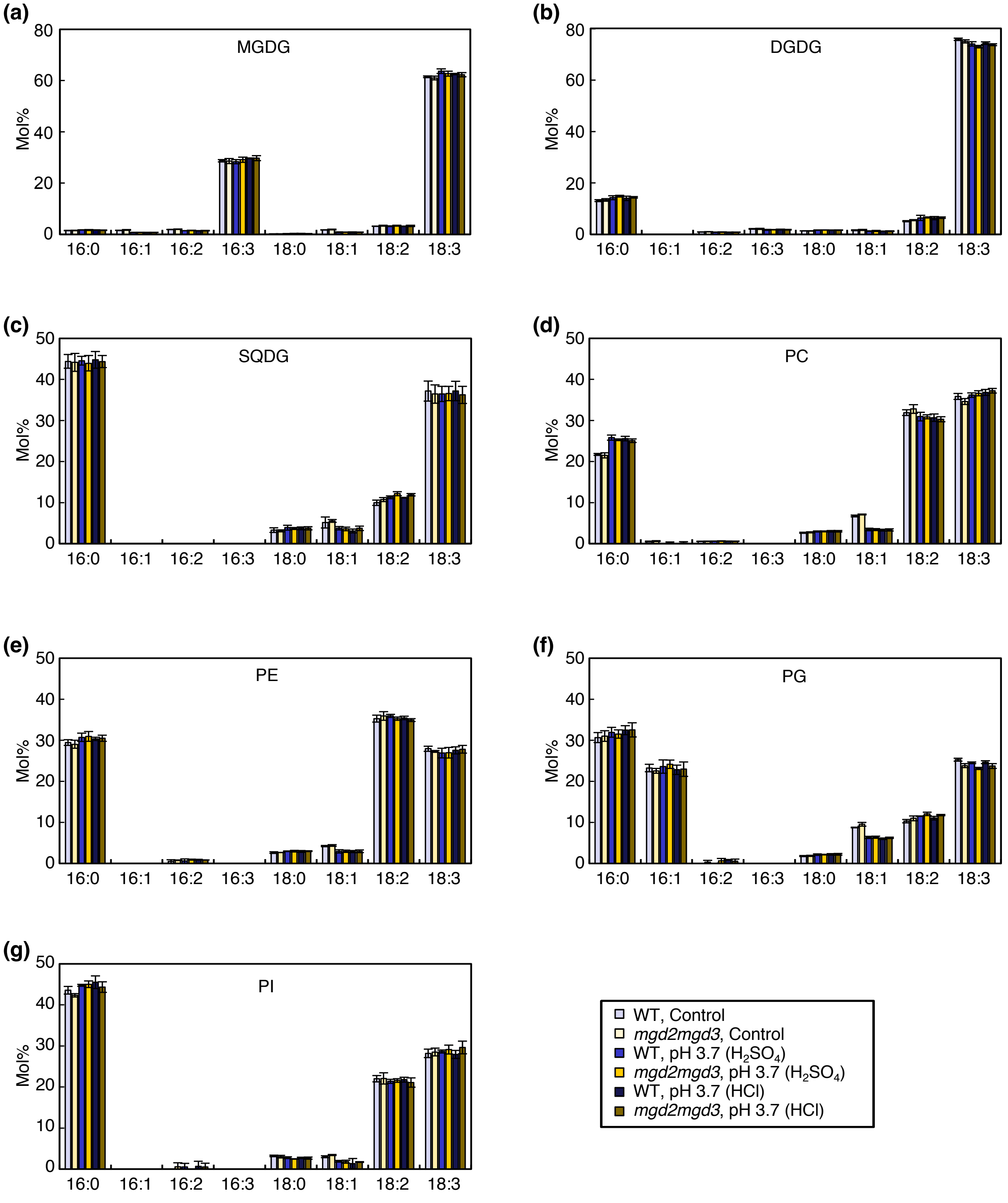


**Figure S1** Fatty acid composition in shoots from WT *Arabidopsis* and *mgd2mgd3* mutant plants grown under acidic conditions. Seedlings were germinated and grown as in Figure 1. (a) to (g) Fatty acid composition of MGDG (a), DGDG (b), SQDG (c), PC (d), PE (e), PG (f) and PI (g) was measured. Values are the mean ± SD ($n = 3$).

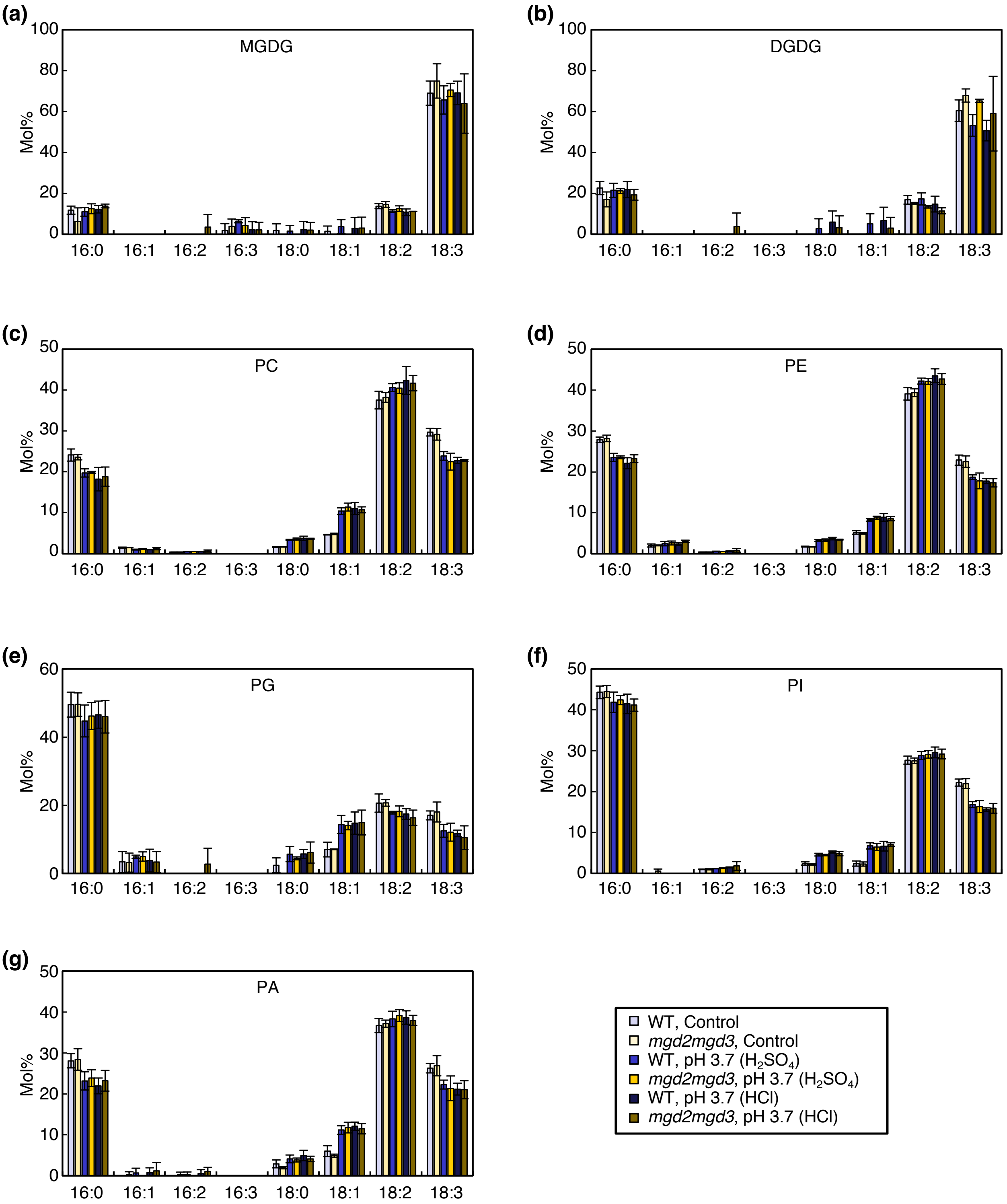


**Figure S2** Fatty acid composition in roots from WT *Arabidopsis* and *mgd2mgd3* mutant plants grown under acidic conditions. Seedlings were germinated and grown as in Figure 1. (a) to (g) Fatty acid composition of MGDG (a), DGDG (b), PC (c), PE (d), PG (e), PI (f) and PA (g) was measured. Values are the mean ± SD ($n = 3$).

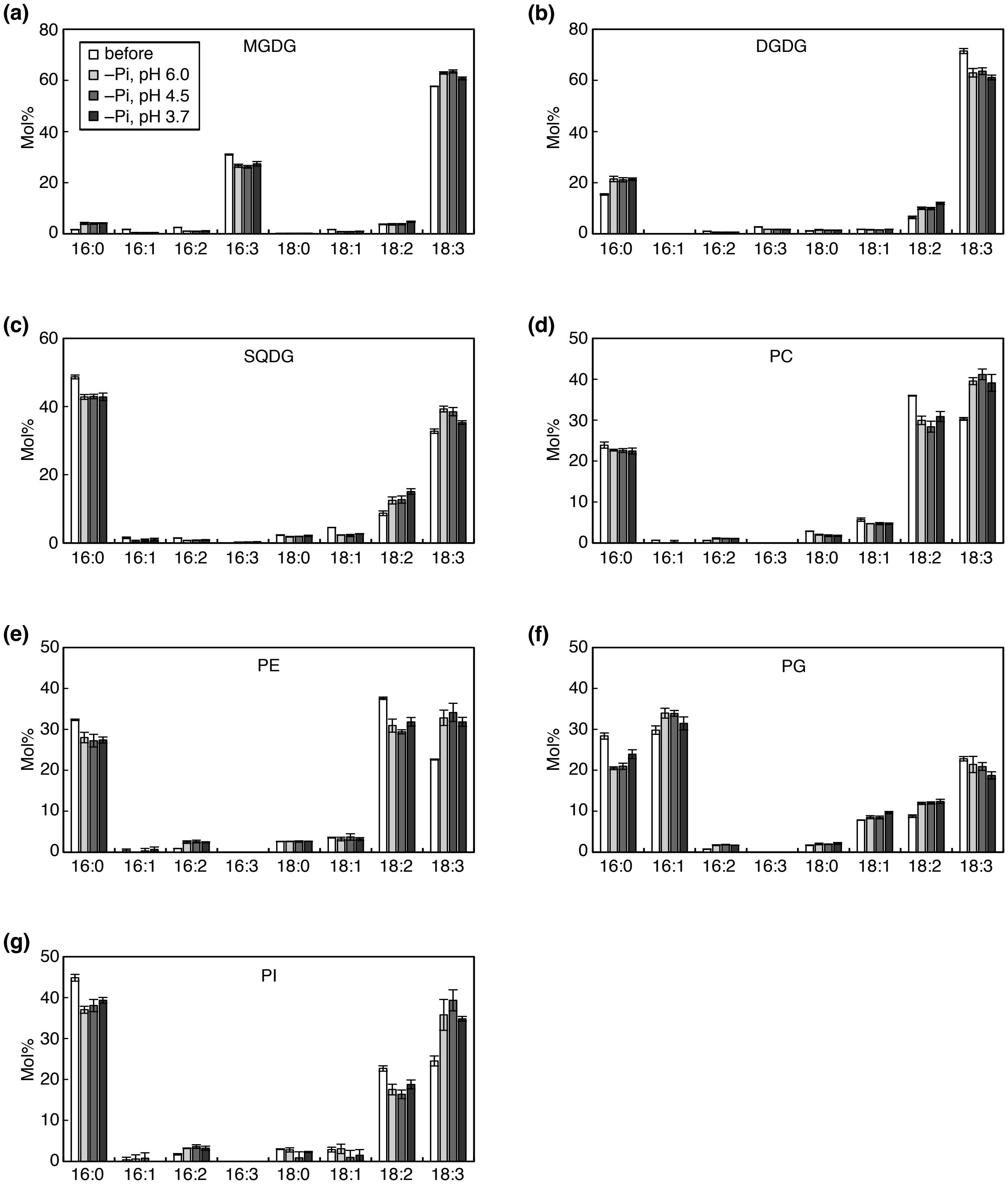


**Figure S3** Fatty acid composition in shoots from WT *Arabidopsis* grown under acidic Pi deficiency. Seedlings were germinated and grown as in Figure 4. (a) to (g) Fatty acid composition of MGDG (a), DGDG (b), SQDG (c), PC (d), PE (e), PG (f) and PI (g) were measured at before (–Pi, 5 d) and after (–Pi, 12 d) a week of acidic Pi depletion stress treatments. Values are the mean ± SD ($n$ = 3).

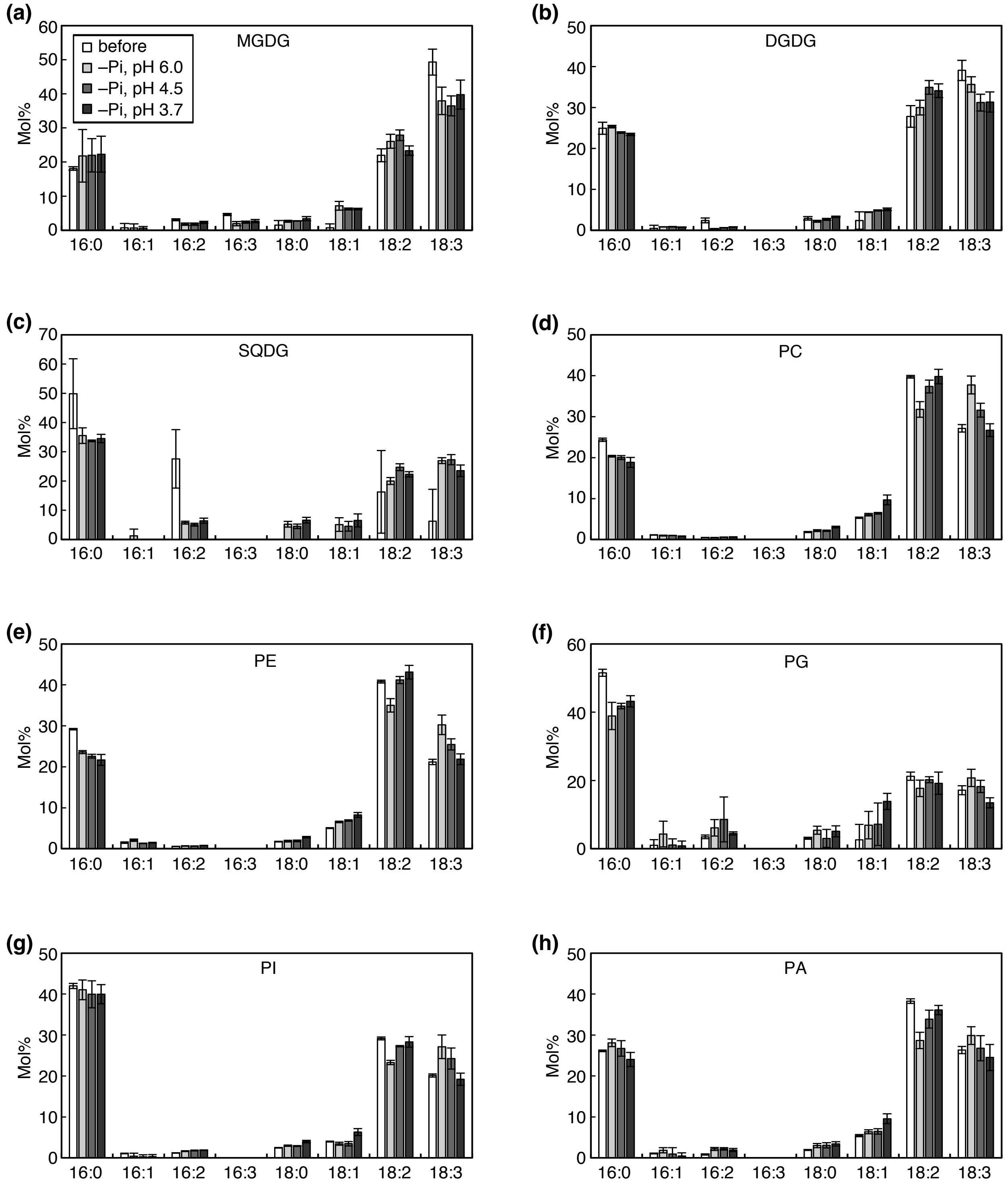


**Figure S4** Fatty acid composition in roots from WT *Arabidopsis* grown under acidic Pi deficiency. Seedlings were germinated and grown as in Figure 4. (a) to (h) Fatty acid composition of MGDG (a), DGDG (b), SQDG (c), PC (d), PE (e), PG (f), PI (g) and PA (h) were measured before (–Pi, 5 d) and after (–Pi, 12 d) a week of acidic Pi depletion stress treatments. Values are the mean ± SD (*n* = 3).

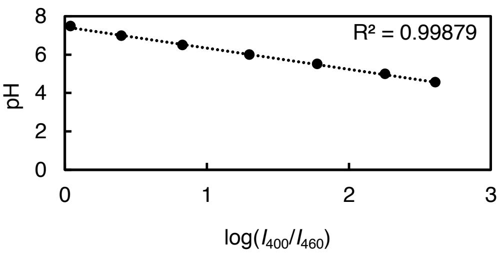


**Figure S5** Calibration curve showing relationship between solution pH and the emission intensity of pyranine. Pyranine emission fluorescence at 510 nm was measured at pH 4.5, 5.0, 5.5, 6.0, 6.5, 7.0 and 7.5 after excitation at 460 and 400 nm. Then, the pH values were plotted relative to the values for the logarithm of the ratio of the emission intensity (*I*) at 400 nm to that at 460 nm.

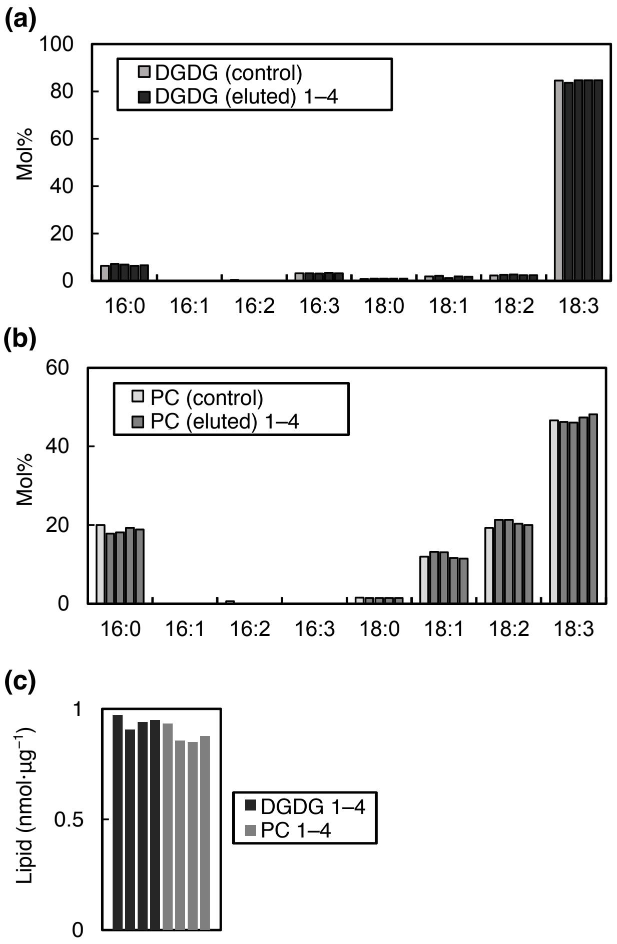


**Figure S6** Evaluation of eluted DGDG and PC obtained from spinach. Fatty acid composition of DGDG (a) and PC (b), which were eluted from silica gel as in Liposome Preparation, was analyzed (four independent extractions are shown). Control was measured as in Lipid Analysis using total lipid from spinach. (c) Lipid content of eluted DGDG and PC.
