# Supplementary material for: Lipid remodeling under acidic conditions and its interplay with low Pi stress in Arabidopsis
Source: Plant Mol Biol. 2019 Jun 14;101(1):81–93. doi: 10.1007/s11103-019-00891-1 (PMC6695348; doi:10.1007/s11103-019-00891-1)
Supplement: Supplementary file 3 — Supplementary material 3 (PDF 129 kb) [file 11103_2019_891_MOESM3_ESM.pdf]

Table SI. Ion concentrations in plant growth medium estimated with GEOCHEM-EZ. Values indicate the percentage of ion complexes in 1/3 MGRL at pH 6.0 and pH 3.5.

Metal	pH 6.0	pH 3.5	
Na (764 μM)	100%	100%	as a free metal
K (1000 μM)	100%	100%	as a free metal
Ca (667 μM)	96%	98%	as a free metal
	1%	1%	complexed with PO_4
	1%	1%	complexed with SO_4
	2%	0%	complexed with EDTA
Mg (500 μM)	98%	99%	as a free metal
	1%	0%	complexed with PO_4
	1%	1%	complexed with SO_4
Fe^{2+} (2.87 μM)	1%	44%	as a free metal
	0%	5%	complexed with PO_4
	99%	50%	complexed with EDTA
Co^{2+} (43 nM)	0%	1%	as a free metal
	100%	99%	complexed with EDTA
Cu^{2+} (0.33 μM)	100%	100%	complexed with EDTA
Zn (0.33 μM)	100%	100%	complexed with EDTA
Mn^{2+} (3.43 μM)	3%	65%	as a free metal
	0%	1%	complexed with SO_4
	97%	34%	complexed with EDTA

Ligand	pH 6.0	pH 3.5	
Cl (87 nM)	100%	100%	as a free ligand
NO_3 (2333 μM)	100%	100%	as a free ligand
PO_4 (590 μM)	1%	1%	complexed with Ca
	1%	0%	complexed with Mg
	98%	99%	complexed with H^+
SO_4 (507 μM)	97%	95%	as a free ligand
	2%	2%	complexed with Ca
	1%	1%	complexed with Mg
	0%	1%	complexed with H^+
$B(OH)_4$ (10 μM)	100%	100%	complexed with H^+
MoO_4 (50 nM)	99%	25%	as a free ligand
	1%	75%	complexed with H^+
EDTA (22 μM)	68%	1%	complexed with Ca
	1%	0%	complexed with Mg
	13%	7%	complexed with Fe^{2+}
	2%	2%	complexed with Cu^{2+}
	2%	1%	complexed with Zn
	15%	5%	complexed with Mn^{2+}
	0%	84%	complexed with H^+

Table SII. Ion concentrations in Pi-depleted growth medium estimated with GEOCHEM-EZ. Values indicate the percentage of ion complexes in 1/3 MGRL –Pi at pH 6.0 and pH 3.5.

Metal	pH 6.0	pH 3.5	
Na (764 μM)	100%	100%	as a free metal
K (1000 μM)	100%	100%	as a free metal
Ca (667 μM)	96%	99%	as a free metal
	1%	1%	complexed with SO_4
	2%	0%	complexed with EDTA
Mg (500 μM)	99%	99%	as a free metal
	1%	1%	complexed with SO_4
Fe^{2+} (2.87 μM)	1%	46%	as a free metal
	99%	53%	complexed with EDTA
Co^{2+} (43 nM)	0%	1%	as a free metal
	100%	99%	complexed with EDTA
Cu^{2+} (0.33 μM)	100%	100%	complexed with EDTA
Zn (0.33 μM)	100%	100%	complexed with EDTA
Mn^{2+} (3.43 μM)	3%	65%	as a free metal
	0%	1%	complexed with SO_4
	97%	34%	complexed with EDTA

Ligand	pH 6.0	pH 3.5	
Cl (87 nM)	100%	100%	as a free ligand
NO_3 (2333 μM)	100%	100%	as a free ligand
SO_4 (507 μM)	97%	95%	as a free ligand
	2%	2%	complexed with Ca
	1%	1%	complexed with Mg
	0%	1%	complexed with H^+
$B(OH)_4$ (10 μM)	100%	100%	complexed with H^+
MoO_4 (50 nM)	99%	25%	as a free ligand
	1%	75%	complexed with H^+
EDTA (22 μM)	68%	1%	complexed with Ca
	1%	0%	complexed with Mg^{2+}
	13%	7%	complexed with Fe^{2+}
	2%	2%	complexed with Cu^{2+}
	2%	1%	complexed with Zn
	15%	5%	complexed with Mn^{2+}
	0%	84%	complexed with H^+

Table SIII. Primer sequences for qPCR.

Gene		Primer sequence	Reference
MGD1	Fw	5' AGGTTTCACTGCGATAAAGTGGTT 3'	Murakawa et al., 2014
	Rv	5' AACGGCAATCCCTCCTCAC 3'	
MGD2	Fw	5' GATTCGATCACTTCCTATCATCCTC 3'	Murakawa et al., 2014
	Rv	5' TGTGCTAAACCATTCCCCAAC 3'	
MGD3	Fw	5' TCGTGGCGGATTGGTTTAG 3'	Murakawa et al., 2014
	Rv	5' CGTTGTTGTTGTTGGGATAGATG 3'	
DGD1	Fw	5' CTGAAGAGAGATCCCGTGGTG 3'	Narise et al., 2010
	Rv	5' TCCCAAGTTCGCTTTTGTGTT 3'	
DGD2	Fw	5' TGCAGAACCTATGACGATGGA 3'	Narise et al., 2010
	Rv	5' GCTCTGTAAGTTGCGATGGTTG 3'	
NPC4	Fw	5' AGCATCAAATGCTGCTGCTCAACC 3'	This study
	Rv	5' TCCACCCACACACAAGAGAAGTGA 3'	
NPC5	Fw	5' CTGCGGTTATGAACGGATTT 3'	This study
	Rv	5' TCGTTGTTCCGTGTGATGTT 3'	
PAH1	Fw	5' GGATAACGAGGACAGGAAGACTG 3'	This study
	Rv	5' AGCAGCTGCGCTAAGTCCCATAC 3'	
PAH2	Fw	5' CTCAAGCCTCAGTCACAAGACAA 3'	This study
	Rv	5' AAGGAAAGAGACCATCAGGAGAGA 3'	
At4	Fw	5' CTGAAGCTCAAGAACCCTCTGAA 3'	Bari et al., 2006
	Rv	5' CCTCTCAAAACCCTTTATTGGTGA 3'	
IAA1	Fw	5' TGAAAGGATCCGAAGCTCCTACT 3'	Inoue et al., 2016
	Rv	5' TGCCTCGACCAAAAGGTGTT 3'	
UBQ10	Fw	5' CCCTAACGGGAAAGACGATTAC 3'	Murakawa et al., 2014
	Rv	5' AAGAGTTCTGCCATCCTCCAAC 3'	
